# Defects in Ribosomal Protein Genes Cause Cancer in Zebrafish

**DOI:** 10.1371/journal.pbio.0020159

**Published:** 2004-05-11

## Abstract

xx

To investigate the genetic underpinnings of a particular biological process, geneticists screen large collections of mutant organisms to characterize their physical defects. By comparing the genetic makeup of nonmutant (called wild-type) organisms to mutants, it's possible to tease out the genes responsible for a defective appearance, or phenotype. In a classic study in the fruitfly, Christiane Nüsslein-Volhard and Eric Weischaus bred many lines of flies with mutations that were lethal: the fly embryos died, but not before displaying a wide range of developmental defects. Since it was known that the fruitfly needed only a single wild-type copy of these genes to survive, the mutations in these “embryonic lethals” had to be recessive, meaning that both copies, or alleles, of the gene had to be mutated for the lethal defect to appear. Nüsslein-Volhard and Weischaus's work revealed many such recessive genes crucial to early development and earned them a Nobel Prize.[Fig pbio-0020159-g001]

**Figure pbio-0020159-g001:**
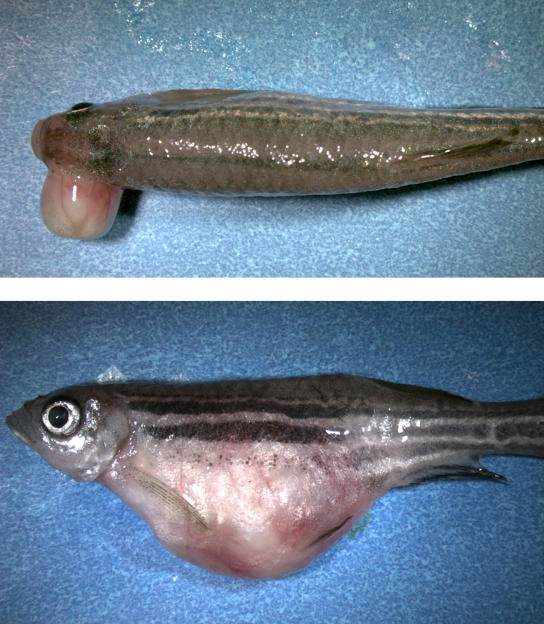
Zebrafish tumors caused by mutation of a ribosomal protein gene

Among the model systems for studying development, the zebrafish has become prized because its transparent embryo develops outside the mother's body. The zebrafish has helped biologists identify many genes involved in embryogenesis and, because it's a vertebrate animal, has become a valuable resource for identifying genes involved in human disease. Now, a team led by Nancy Hopkins of the Massachusetts Institute of Technology, has created over 500 lines of zebrafish with lesions in key embryogenic genes and used them to identify a group of genes that predispose the fish to cancer, with some surprising results.

All of the 500 lines created by the researchers carried a recessive embryonic lethal mutation; for about 400 of the lines, mutations in 300 distinct genes were identified as the cause of the embryonic phenotype. During the process of cultivating some of these mutant lines, the Hopkins team noticed that an abnormally large percentage of fish experienced early mortality (in some cases, over 50% compared to the 10%–15% seen in nonmutant fish), while the surviving fish in these lines developed large, highly invasive malignant tumors; both phenotypes persisted over successive generations. The tumors resembled malignant peripheral nerve sheath tumors (MPNSTs) that have been found in other fish species as well as in mammals. Suspecting that these mutant lines had elevated rates of cancer, the researchers investigated the genetic makeup of the fish and discovered to their surprise that each line was heterozygous for a mutation in a different ribosomal protein gene *(rp)*—that is, each line carried one healthy version and one defective version of a different *rp* gene. These proteins are components of ribosomes—the massive molecular complexes within cells that mediate protein synthesis—and are essential for embryonic development.

All of the *rp* mutations, the researchers report, either reduced or eliminated expression of the corresponding *rp* gene. In the case of “classic” tumor suppressor genes, the wild-type allele must be lost for the defective allele to set the stage for cancer. Here, the wild-type allele appeared to remain intact in the tumor cells, implicating the proteins as “haploinsufficient” tumor suppressors—a reduction from two gene copies to one functional copy seems to be enough to increase the risk of cancer. Apart from the mutations in *rp* genes, the authors also found a loss-of-function mutation in a gene (called *NF2*) that acts as a tumor suppressor in mammals—establishing the soundness of this approach for identifying mammalian cancer genes.

While these experiments do not explore how these mutations lead to cancer, the results suggest that some shared, ribosome-associated function allows these genes to act as tumor suppressors and that disrupting this function somehow leads to tumor formation. Though it's not clear what distinguishes the 11 *rp* genes whose mutations caused cancer from the five other *rp* genes whose mutations did not, the authors raise a number of possibilities for future study. And given the high degree of conservation of genes and pathways among vertebrates, it's likely that *rp* mutations also raise cancer risk in humans. Together, these results demonstrate that the tiny freshwater workhorse of developmental biology has a promising future as a model system for human cancer.

